# Passive flow-field control using dimples for improved aerodynamic flow over a wing

**DOI:** 10.1038/s41598-024-63638-z

**Published:** 2024-06-05

**Authors:** Haris Ali, Mohammad Rasidi Rasani, Zambri Harun, Muhammad Ashhad Shahid

**Affiliations:** 1 Department of Mechanical and Manufacturing Engineering, Faculty of Engineering and Built Environment, Universiti Kebangsaan Malaysia (UKM), 43600 Bangi, Selangor Malaysia; 2 Department of Basic Sciences, DHA Suffa University, Karachi, Sindh 75500 Pakistan

**Keywords:** Aerodynamics, CFD, RANS, Turbulence, Passive flow control, Dimples, Boundary layer, Engineering, Aerospace engineering, Mechanical engineering

## Abstract

This study explores the efficacy of dimples in influencing the aerodynamic performance of a straight rectangular wing. Computational Fluid Dynamics based numerical simulations were performed to model turbulent flow and quantify the forces exerted on the wing. The *k-ω* Shear-Stress Transport turbulence model was chosen to solve the underlying equations. To ascertain reliability, the results of numerical simulations were compared with both experimental and simulation results of the previous studies. The impact of various dimple configurations, placed at 15%, 50% and 85% of the chord length, on the aerodynamic performance of the wing was investigated. The evaluation involved analyzing the drag coefficient (*C*_*D*_), lift coefficient (*C*_*L*_), lift-to-drag (*L/D*) ratio, streamlines and the flow field around wing in both chordwise and spanwise directions. The findings indicated that a wing with a dimpled surface could yield a reduced drag coefficient of up to 6.6% compared to the unmodified wing. This reduction is attributed to the dimples ability to sustain attached airflow and delay flow separation. The results demonstrated negligible deviation in the lift coefficient with the incorporation of dimples. The incorporation of dimples on the wing surface has been demonstrated to enhance the aerodynamic performance of lifting surfaces.

## Introduction

The growing interest in turbulent flow control is driven by significant economic and environmental considerations. Reducing drag caused by fluid motion around a solid object is not only a fundamental exploration of optimizing the interaction with turbulence but also a significant technological challenge with wide-ranging applications, driven by economic and environmental considerations. One of the most important applications is the aerospace industry where the drag produced by an aircraft is one of the major impediments to its performance. Aircraft facing less drag will require less power saving the fuel, thus making flight, commercial, more efficient and economical.

In scenarios featuring planar walls, the entirety of the drag is attributed to skin-friction drag, which emerges as a pivotal outcome of turbulence's dissipative nature. Therefore, in such configurations, skin-friction drag constitutes 100% of the total drag^1–3^. For large-scale engineering applications including aerospace industry, the viscous drag causes as much as 50% of the total drag^4^. Several studies have explored the design trade-offs to optimize the aerodynamic performance of flying bodies. In general, the aerodynamic performance of the airplane wings can be optimized by incorporating the surface flow control techniques which can effectively reduce the skin friction drag^5^.

These techniques can be categorized into active and passive methods. Active control strategies are preferable for their adaptability in diverse operational conditions. However, these methods involve increased financial outlays, demanding supplementary investments in energy provision, controllers, and air systems, consequently restricting their applicability. In contrast, passive flow control techniques center around adding supplementary structures to the body or making slight modifications to the body's geometrical shape. Passive strategies, being simpler and more cost-effective, are considered more practical than the active flow control methods. Therefore, in the pursuit of improving the aerodynamic performance of lifting surfaces, passive control methods are preferred. These passive techniques are utilized to influence stall phenomena or delaying the flow separation without the need for external energy consumption^6^.

Among the passive flow control, the use of winglets^7–15^ at the wingtips is a most common method, but it is typically used to reduce the induced drag caused by the downwash of wing-tip vortices. Another prominent passive technique is the use of riblets^16–21^. NASA introduced riblets in the 1980s, and their potential in aviation applications has been widely researched in the years thereafter. Characterized by streamwise-aligned microgrooves, riblets have demonstrated the ability to effectively diminish friction drag. Riblets can take on various cross-sectional shapes, with the triangular profile being particularly popular. However, a crucial characteristic is the presence of a very sharp tip. Despite recent advancements^22,23^ indicating a promising future for riblets in aeronautics with potentially improved cost–benefit ratios, their deployment in commercial transport aircraft is presently constrained. This is attributed to limited cost savings^24,25^ and significant manufacturing and maintenance challenges arising from the miniscule dimensions of riblets and the necessity to maintain their tips in a sharp condition.

A recent alternative to riblets, which is easier to manufacture and lacks sharp details, has emerged. This alternative involves impressing a pattern of small dimples onto the surface. Dimples, referring to small concavities imprinted on a surface, have undergone extensive study in the past due to their capability to enhance surface heat transfer^26^. The utilization of dimples on bluff bodies, like golf balls, is widely acknowledged for its influence on the turbulent boundary layer and separation^27^. This approach is also being explored in the context of sports car racing^28^.

Dimples function as a surface roughness, promoting a turbulent boundary layer and consequently causing delayed flow separation, a diminished wake, and reduced form drag. Nevertheless, incorporating a dimple on a streamlined object, like an airfoil at different angles of attack, may contribute to delaying flow separation and minimizing wake size. However, it could potentially result in an increased friction drag, presenting a trade-off^29^. Asai et al.^30^ conducted experimental wind tunnel testing to test volleyball balls with dimple and honeycomb surfaces. Their findings revealed that the critical Reynolds number was slightly lower for the new balls. Honeycomb balls had a higher drag coefficient than conventional ones, and dimpled balls showed greater variances in flight orientation. Joseph et al.^31^ conducted numerical research on the impact of square dimples on the suction side of the NACA0012 airfoil wing at various angles of attack. Their conclusion highlighted that the dimples have the potential to delay the stall angle.

In an experimental investigation, Tay et al.^32^ studied the impact of circular axisymmetric and teardrop-shaped dimples on drag reduction in a turbulent channel flow for Reynolds numbers ranging from 5000 to 50,000. The highest drag decrease was recorded when the sharp tip directed upstream rather than downstream. Additionally, they observed that the drag reduction of teardrop-shaped dimples surpassed that of circular axisymmetric dimples under the same conditions. Lu et al.^33^ numerically investigated the influence of dimples on aerodynamic performance in linear compressor cascades, specifically analyzing the impact of dimple location on flow characteristics and loss behavior through three different configurations. Their findings contributed valuable insights to the field of dimple flow control, offering guidance for optimizing the design of linear compressor cascades.

Zhang et al.^34^ investigated on drag reduction in marine vessels, employing both experimental and computational approaches. The study focused on the integration of air-filled dimples and their optimization in terms of shape and size on the vessel surfaces. Their conclusion emphasized that excessively increasing the air volume results in counterclockwise air swirl downstream, suggesting that larger dimples may not always be optimal. In scenarios without air injection, properly designed dimple geometries could achieve a drag reduction of up to 40.38%. Wang et al.^35^ computationally reduced drag on a vehicle body using a dimpled non-smooth surface, resulting in altered drag coefficients by minimizing turbulent kinetic energy and wake vortices. Chear and Dol^36^ conducted numerical analyses to study the impact of a different depth to diameter ratios of dimples on the drag coefficient of a car model. Their findings suggested that each evaluated dimple ratio contributed to reducing the drag coefficient by a maximum of 1.95%.

Utilizing dimples to delay boundary layer separation and mitigate pressure drag force represents a plausible hypothesis for enhancing the aerodynamic performance of lifting surfaces, such as the wings of an aircraft.

Therefore, this study numerically analyzes the impact of incorporating surface dimples on the aerodynamic performance of a straight rectangular wing. To achieve this objective, the wing surfaces are modified passively by incorporating spherical dimples. These dimples are strategically positioned at various chord lengths with specific pitch distances between them. The governing equations, which encompass continuity and momentum, are solved through the utilization of an incompressible Reynolds-Averaged Navier–Stokes (RANS) solver coupled with the *k-ω* SST turbulent model. The impact of dimples on the aerodynamic performance of the wing was evaluated in terms of the drag coefficient, lift coefficient, L/D ratio, pressure contours and streamlines, and the flow field analysis. The findings indicate that dimples have the potential to be effective in minimizing drag and enhancing the L/D ratio if they are designed appropriately.

The organization of this article is as follows: Section “Problem description” provides details on the wing with dimples design configurations. Section “Governing equations and boundary conditions” offers a concise explanation of the computational framework and simulation methodology employed in this study. Section “Meshing and validation” delves into the meshing process and its validation based on previous studies. Section “Results and discussion” presents the findings and corresponding discussions, followed by the conclusion in Section “Conclusion”.

## Problem description

In this investigation, a CFD approach is employed to examine the aerodynamic performance of a wing featuring dimples. With the swift progression of computational capabilities, CFD has become a preferred tool in diverse engineering disciplines. This preference is attributed to its capability in effectively resolving the Navier–Stokes equations for intricate problems related to fluid–fluid and fluid–solid interaction, relying on the fundamental laws of conserving mass, energy and momentum. This is achieved by employing various turbulence models under diverse operating conditions. Furthermore, CFD enables the generation and examination of diverse output results during post-processing. This encompasses, yet is not restricted to, the visualization of flow streamlines, pressure, temperature and velocity contours, flow animations, and other informative representations of the solution. These high quality visuals play a crucial role in elucidating findings, as they enhance the understanding of intricate flow processes.

**Figure 1:**
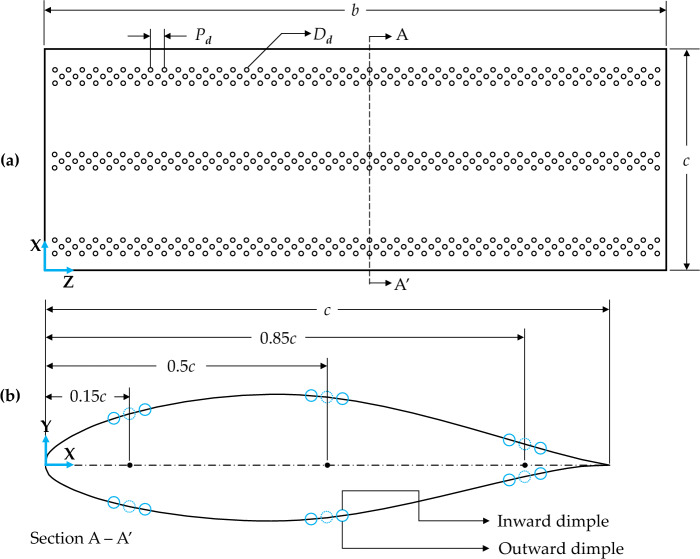
(**a**) 2-D top view of a dimpled wing; (**b**) Cross-section A—A′ of dimpled airfoil.

As previously mentioned, this study numerically investigates the impact of surface dimples on the aerodynamic performance of a straight rectangular wing. The results of the baseline wing are validated with the available findings from computational studies and experiments in existing literature. Then the baseline wing is modified by placing three rows of spherical dimples each at 15%, 50% and 85% of the chord length starting from the leading edge to the trailing edge as shown in Fig. 1. A total of six configurations are examined; configurations 1 to 3 consisted of inward dimples on suction side, pressure side and on both sides respectively, configurations 4 to 6 consisted of outward dimples in the same manner as explained in Table 1. The dimples in this study have a pitch distance, *P*_*d*_, of 1.5D_d_, and a dimple diameter, *D*_*d*_, of 0.02*c* where *c* represents the wing’s chord length. The midpoint of spherical dimples is accurately placed on the wing’s surface (or along the airfoil edge in a 2D view) to maintain a dimple depth equal to 0.075*D*_*d*_. The simulations are performed for low subsonic free stream velocity of 32.15 m/s which corresponds to the Reynold number $\left(\text{Re}=\frac{\rho VL}{\mu }\right)$ of 2.5 × 10^5^, where the chord length, *c*, has been used as characteristic length in *Re* calculation. The geometric characteristics of the studied dimple configurations and flow parameters are summarized in Table 2.

**Table 1:** Description of studied dimpled wing configurations.

Shape at cross-section A—A′	Nomenclature	Configuration description
	C1-IDSS	Inward dimples on suction surface
	C2-IDPS	Inward dimples on pressure surface
	C3-IDBS	Inward dimples on both pressure and suction surfaces
	C4-ODSS	Outward dimples on suction surface
	C5-ODPS	Outward dimples on pressure surface
	C6-ODBS	Outward dimples on both pressure and suction surfaces

**Table 2:** Geometric specifications and flow parameters.

Parameter	Definition/value
Airfoil profile	NACA 65_3_–218
Chordlength, *c*	0.121 m
Wing span, *b*	0.66 m
Reference area, *S*	0.08 m^2^
Dimple pitch distance, *P*_*d*_	1.5*D*_*d*_
Dimple diamater, *D*_*d*_	0.02*c*
Dimple depth, *d*	0.075*D*_*d*_
Dimple Layout	Staggered
Free stream velocity, *V*	32.15 m/s
Angle of attack, *α*	0–12° (Δα = 4°)

## Governing equations and boundary conditions

A steady-state and incompressible approach is adopted to simulate the flow around the wing. The study employs an incompressible RANS solver coupled with the *k-ω* SST turbulence model for problem resolution. The governing equations, encompassing continuity and momentum, are represented as follows^37^:

1 $$\frac{\partial {\overline{u} }_{i}}{\partial {x}_{i}}=0$$

2 $$\frac{\partial \left(\rho {\overline{u} }_{i}\right)}{\partial t}+\frac{\partial \left(\rho \overline{{u }_{i}{u}_{j}}\right)}{\partial {x}_{j}}=\frac{\partial \overline{p}}{\partial {x }_{j}}+\frac{\partial }{\partial {x}_{j}}\left(\mu \frac{\partial {\overline{u} }_{i}}{\partial {x}_{j}}-\rho \overline{{u }_{i}{\prime}{u}_{j}{\prime}}\right)$$

Here,

*ū* and *u*′ = average and instantaneous components of velocity, respectively. *p* = pressure. *ρ* = fluid density. *x*_*i*_ and *x*_*j*_ = cartesian coordinates. *t* = time. *µ* = fluid dynamic viscosity.

The *k-ω* SST turbulence model is renowned for combining the capabilities of the *k*—*ω* model in the inward regions of the boundary layer and the *k*—*ε* model in the free stream. The complete equation for this model is outlined below^38^:

3 $$\frac{\partial \left(\rho k\right)}{\partial t}+\frac{\partial \left(pk{u}_{i}\right)}{\partial {x}_{i}}=\frac{\partial }{\partial {x}_{j}}\left({\Gamma }_{k}\frac{\partial k}{\partial {x}_{j}}\right)+{G}_{k}-{Y}_{k}+{S}_{k}$$

4 $$\frac{\partial }{\partial t}\left(\rho \omega \right)+\frac{\partial }{\partial {x}_{i}}\left(\rho \omega {u}_{i}\right)=\frac{\partial }{\partial {x}_{j}}\left({\Gamma }_{\omega }\frac{\partial \omega }{\partial {x}_{j}}\right)+{G}_{\omega }-{Y}_{\omega }+{D}_{\omega }+{S}_{\omega }$$

Here,

*k* = turbulence kinetic energy. *ω* = specific heat dissipation rate. *G*_*k*_ = generation of turbulence kinetic energy due to average velocity gradients. *G*_*ω*_ = generation of *ω*. *Г*_*k*_ and *Г*_*ω*_ = effective diffusivity of *k* and *ω*, respectively. *Y*_*k*_ and *Y*_*ω*_ = dissipation of *k* and *ω* due to turbulence. *D*_*ω*_ = cross-diffusion term. *S*_*k*_ and *S*_*ω*_ = user-defined source terms.

**Figure 2:**
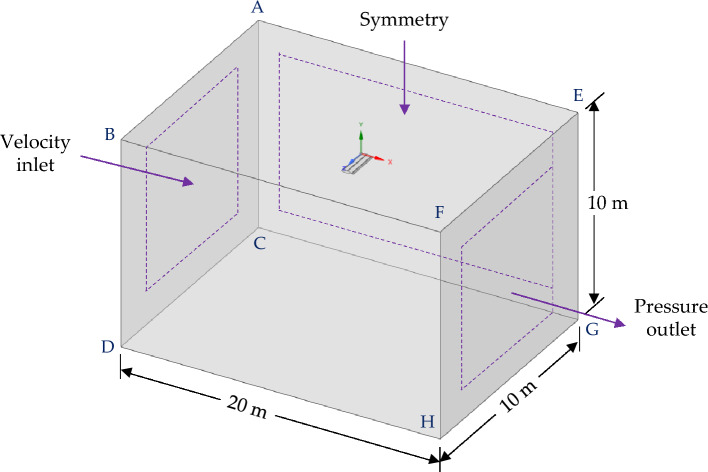
Illustration of boundary conditions with computational domain.

This research is numerically conducted using ANSYS Fluent within a fixed (inertial) reference frame (FRF). The core principle of the FRF is the assumption that the coordinate system remains constant and does not move with the flow. This is commonly used for studying external aerodynamic flows, such as the flow around an aircraft or a wing. The boundary conditions with geometry enclosed within the domain are illustrated in Fig. 2. In this simulation, wing with dimples is modelled and put inside a fixed rectangular enclosure for considering the wind field. The size of the rectangular domain is 20 m × 10 m × 10 m, which is dictated by the chord length, c, of the wing. A domain size of approximately 10 to 20 chord lengths upstream and 20 to 40 chord lengths downstream is considered appropriate for subsonic wing simulations.

The assumption of steady-state and incompressible airflow is employed in the simulations. A velocity inlet boundary condition is established at the fixed enclosure's inlet (rectangular domain), representing a free stream velocity of 32.15 m/s with 5% turbulent intensity. Meanwhile, a pressure outlet boundary condition is set at the exit of the fixed enclosure, where the pressure gauge is maintained at zero. The rightmost part of the fixed enclosure is assigned a symmetry boundary condition, considering a zero side-slip angle for all test cases. The wing surface is subjected to a no-slip wall condition. The solver operates on a pressure-based formulation with a relative velocity approach. The coupling between pressure and velocity is addressed through the use of the COUPLED scheme. For both pressure and momentum equations, a “second-order upwind spatial discretization” algorithm is employed, and the calculation of gradients utilizes a least squares-based cell-centered algorithm. Convergence criteria for all residuals are set to 10^–6^.

The aerodynamic performance of aircraft or a wing is usually measured in terms of lift coefficient (*C*_*L*_) and drag coefficient (*C*_*D*_). Lift coefficient represents the lift force (*L*) generated by the wings, while drag coefficient represents the drag force (*D*) that opposes the forward motion of the aircraft. Lift coefficient is used to calculate the aerodynamic loads on an aircraft during flight. These loads are important for structural design and ensuring that the aircraft's components can withstand the forces they will encounter. Reducing drag is essential for improving an aircraft's fuel efficiency. Lower drag means lower engine power requirements to maintain a specific airspeed, thereby leading to decreased fuel consumption. These two aerodynamic parameters are defined as follows^39^:

5 $${C}_{L}=\frac{L}{0.5\rho {V}_{\infty }^{2}S}$$

6 $${C}_{D}=\frac{D}{0.5\rho {V}_{\infty }^{2}S}$$

Here, *V*_*∞*_ = free stream velocity. *S* = reference area of the wing.

## Meshing and validation

**Figure 3:**
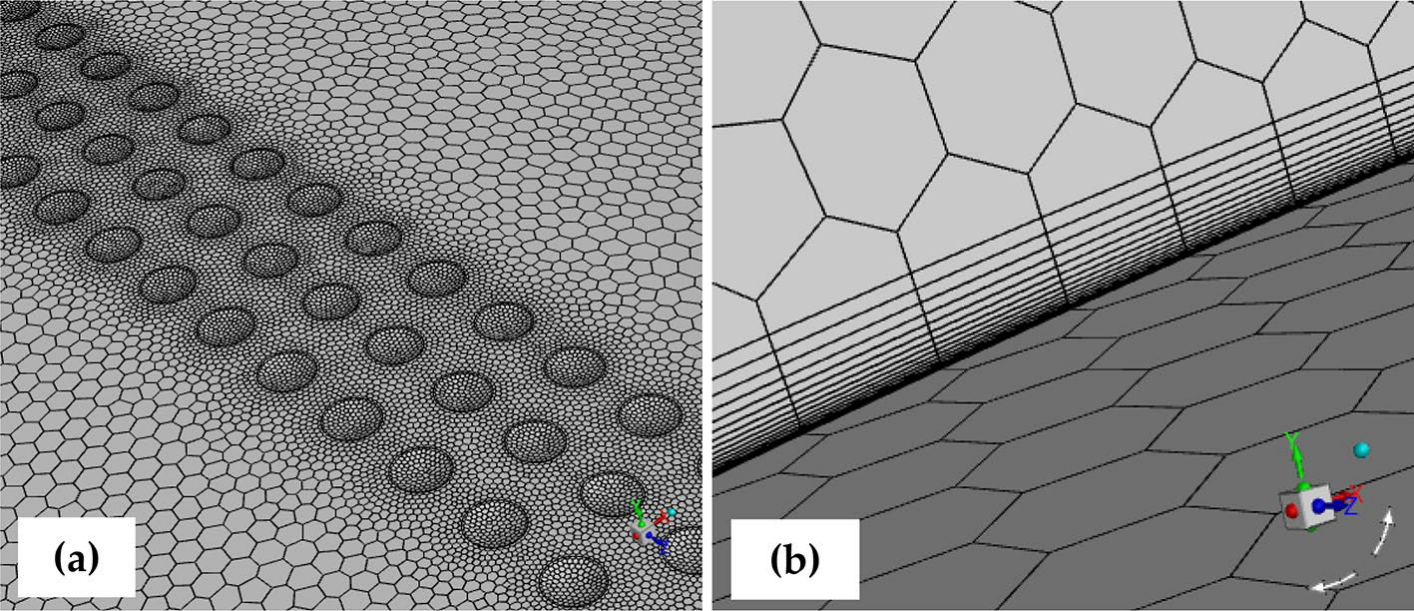
Mesh implementation; (**a**) Dimpled wing; (**b**) Inflation layers to capture boundary layer.

In this study, an unstructured mesh is chosen for domain discretization. The mesh quality is ensured with an orthogonal quality exceeding 0.152 and a skewness kept below 0.558. Node concentration is strategically increased at the leading edge to capture significant pressure gradients and near the dimples for precise representation of their impact. The dimple face size is 0.001 m, approximately ten times smaller than the size of the rest of the wing surface. This size has been found to be sufficient for adequately resolving flow features within the dimples, ensuring accurate simulation results. Following a comprehensive mesh independence study, the total mesh element count is determined to be around 7.9 million. Figure 3a illustrates the application of the surface mesh on the wing and near the dimples, while Fig. 3b provides a visual representation of inflation layers for capturing the boundary layer. In turbulent flow simulations using the *k-ω* SST turbulent model, it is advisable to maintain the y + parameter below 3 to precisely depict the boundary layer^40^. The initial step in determining the y + value involves calculating the height of the first layer of inflation. This is achieved by applying equations based on the flat plate flow approach, as indicated by the following expressions:

7 $$\Delta y=\frac{{y}^{+}\mu }{\rho {u}^{*}}$$

8 $${u}^{*}=\sqrt{\frac{{\tau }_{\omega }}{\rho }}$$

Here, Δy = height of the first layer. y^+^ = target y + value. *μ* = dynamic viscosity. *ρ* = air density. u^*^ = friction velocity. *τ*_*ω*_ = wall shear stress.

The mesh is generated using the initially estimated height value obtained before the simulation. To achieve this, twenty prism layers are introduced near the walls, with the first layer height of 1 × 10^–5^ m and the growth rate of 1.18. The results demonstrate that the y + parameter is predominantly below one on most of the wing surface. This ensures an adequate number of nodes near the walls, enabling accurate resolution of the viscous sub-layer.

**Figure 4:**
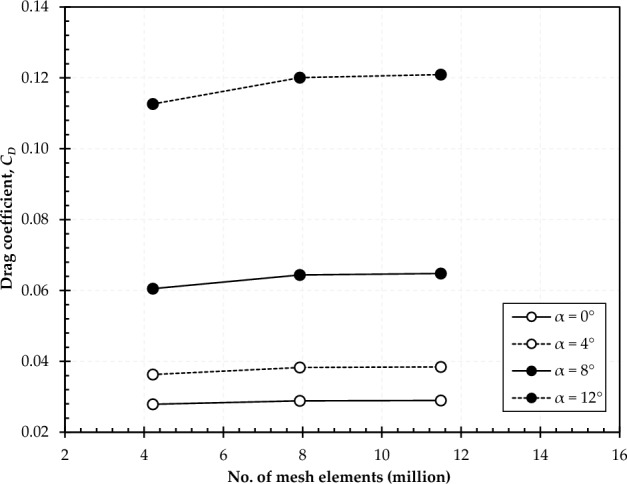
Mesh independence study (drag coefficient, *C*_*D*_) performed on C1-IDSS.

For the grid independence study, a wing modified with inward dimples on the suction side is selected. The drag coefficient is evaluated at all studied angles of attack (0°, 4°, 8°, and 12°) and a free stream velocity of 32.15 m/s, using different numbers of mesh elements, as depicted in Fig. 4. The results presented in Fig. 4 demonstrate that the discrepancy in drag coefficients between the case with 7.93 million elements and the case with the finest mesh (i.e., 11.49 million elements) is less than 0.7% at the highest simulated angle of attack (12°). Consequently, to reduce computational cost and time, we employed the mesh with 7.93 million elements for the subsequent simulations.

**Figure 5:**
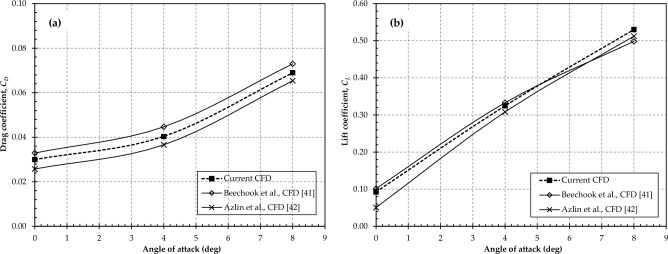
Comparison between current and previous studies; (**a**) Drag coefficient; (**b**) Lift coefficient.

In addition to the mesh independence study, the validity of the present study is established by comparing the *C*_*L*_ and *C*_*D*_ of the baseline wing (without dimples) to CFD results available in the literature. The results are displayed in Fig. 5a and b. It is evident that, in most cases, the deviations are less than 5% when compared to the simulation results of Beechook et al.^41^ and Azlin et al.^42^, which falls within an acceptable range. Notably, the lift and drag curve trends in our present study consistently align closely with those of previous simulations cited above.

To further verify the accuracy of the numerical scheme and mesh resolution used, the classical case of wind tunnel testing results of the ONERA M6 wing has been selected as a standard for validation due to the extensive experimental data available for this particular case. The computed surface pressure distribution is compared with the experimental results of Schmitt et al.^43^. The geometry of the ONERA M6 wing was taken from^44^. The validation case was simulated at α = 3.06°, for which experimental data is available at *Mach* 0.84. Based on the mean aerodynamic chord (*MAC* = 0.64607 m) of the ONERA M6 wing, the flow Re number is 11.7 × 10^6^. The simulation was carried out using the same mesh resolution and solution scheme employed in the current study, utilizing the *k-ω* SST turbulence model while maintaining y + ≈ 1 over the surface of the ONERA M6 wing.

**Figure 6:**
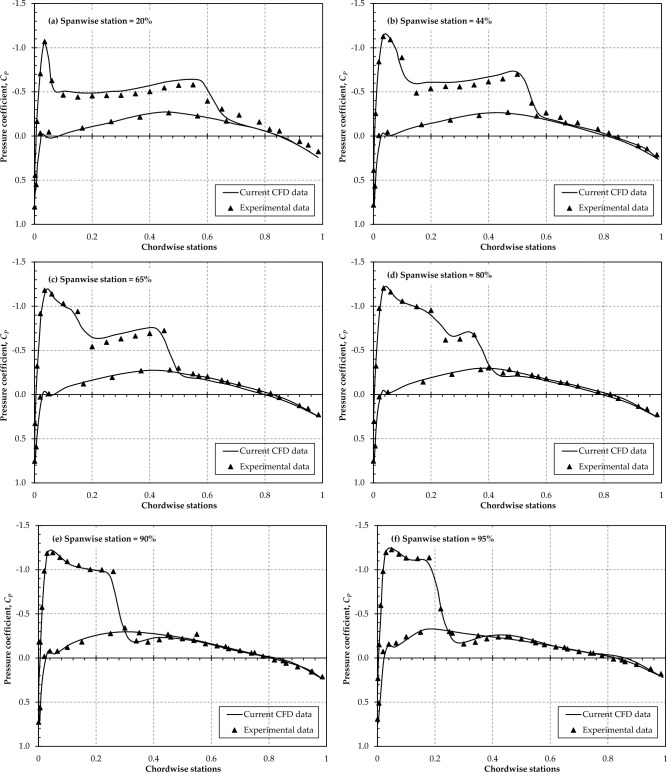
Comparison between experimental ^43^ and current simulated *Cp* distribution for the ONERA M6 wing.

The next step in the validation process is to compare the simulated surface pressure distribution with the experimental results. For this purpose, the pressure coefficient, *Cp* was plotted at six different spanwise locations for which experimental data is available. As evident from Fig. 6, the numerical solution shows a similar trend in the *Cp* distribution as observed in the wind tunnel experiments, and overall, the prediction is accurate.

Based on these results, we can assert that the selected numerical scheme, turbulence model, and mesh resolution are adequate to resolve the physics involved in the present study. These findings demonstrate the precision of the numerical techniques implemented in the software and confirm the accuracy of the present simulations.

## Results and discussion

As outlined in preceding sections, this study conducts a numerical analysis of the impact of introducing dimples on the surface of a straight rectangular wing. Spherical dimples placed in three rows, each with a diameter of *D*_*d*_, are positioned at 15%, 50%, and 85% chordwise from the leading edge. The influence of dimple shape (either inward or outward) and dimple location (suction surface, pressure surface, and both) is studied at a low subsonic flow velocity of 32 m/s for angles of attack ranging from 0 to 12 degrees. The following section presents the numerical results of dimples and their effect on aerodynamic characteristics, along with the visualization of flow fields and surface contours.

### Aerodynamic performance of dimples

**Figure 7:**
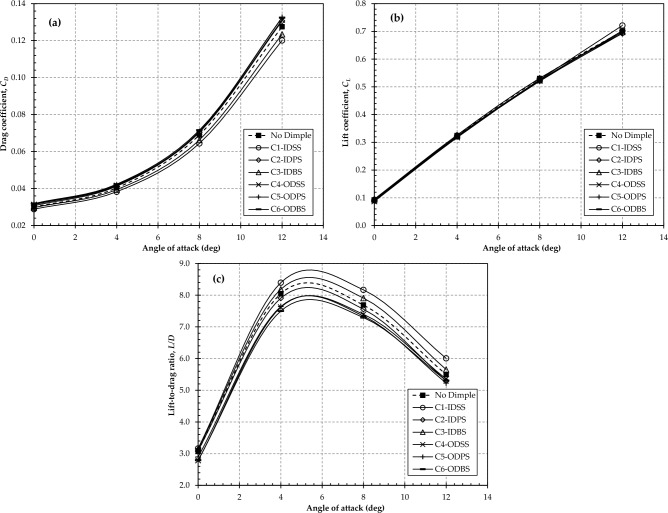
Effect of dimples on aerodynamic characteristics; (**a**) *C*_*D*_; (**b**) *C*_*L*_; (**c**) *L*/*D* ratio.

The impact of dimples on the aerodynamic characteristics of the wing is presented in Fig. 7. The findings are also contrasted with those of the original wing configuration (wing without dimples). It can be observed from Fig. 7a that the drag coefficient of C1-IDSS and C3-IDBS reduced at all studied angles of attack, showing that implementing inward surface dimples on the suction surface yields better performance in terms of drag reduction. However, the drag coefficient for the configurations with outward dimples is increased for all placement surfaces. This indicates that introducing outward dimples does not provide any advantage in terms of the drag coefficient. In contrast, when examining the lift coefficient in Fig. 7b, it is evident that the dimpled wings exhibit no significant deviation in lift coefficient compared to the original wing. This suggests that the introduction of surface dimples may not alter the structural characteristics of the wing, and therefore, no notable cost is anticipated for the structural design of the dimpled wing. Since there is an improvement in drag coefficient with no significant change in lift coefficient, it leads to an improved lift-to-drag ratio for config 1 at all angles of attack, as shown in Fig. 7c.

The Tables 3, 4, and 5 provide a summary of percentage changes (either increase or decrease) in drag coefficient, lift coefficient, and lift-to-drag ratio for all studied dimple configurations. From Table 3, the maximum decrease in the drag coefficient of 6.60% was obtained by C1-IDSS at an 8° angle of attack. Similarly, from Table 5, C1-IDSS shows the optimum aerodynamic performance with an increase in L/D ratio of 9.29% achieved at a 12° angle of attack. Hence, these enhancements imply that integrating dimples can enhance the aerodynamic performance of lifting surfaces when designed and implemented appropriately.

**Table 3:** Percentage change in aerodynamic drag coefficient, *C*_*D*_ due to dimples.

α (°)	Percentage increase (+)/decrease (−) in drag coefficient (%)						
	No Dimple	C1-IDSS	C2-IDPS	C3-IDBS	C4-ODSS	C5-ODPS	C6-ODBS
0	0.00	− 3.82	2.42	− 0.98	4.45	4.96	6.36
4	0.00	− 5.14	2.24	− 2.42	3.20	3.88	4.94
8	0.00	**− 6.60**	1.99	− 3.93	2.34	2.79	3.75
12	0.00	− 5.84	2.43	− 3.23	2.82	3.99	3.82

Bold signifies the maximum drag reduction and L/D ratio enhancement achieved.

**Table 4:** Percentage change in aerodynamic lift coefficient, *C*_*L*_ due to dimples.

α (°)	Percentage increase (+)/decrease (−) in lift coefficient (%)						
	No Dimple	C1-IDSS	C2-IDPS	C3-IDBS	C4-ODSS	C5-ODPS	C6-ODBS
0	0.00	− 1.47	2.74	0.91	− 5.87	− 1.58	− 2.39
4	0.00	− 1.07	0.41	− 0.78	− 2.33	− 1.40	− 1.15
8	0.00	− 0.78	0.24	− 1.20	− 1.55	− 1.85	− 0.80
12	0.00	2.90	− 1.22	− 0.49	0.19	− 1.11	− 2.63

**Table 5:** Percentage change in aerodynamic lift-to-drag ratio, *L/D* due to dimples.

α (°)	Percentage increase (+)/decrease (−) in lift-to-drag ratio (%)						
	No Dimple	C1-IDSS	C2-IDPS	C3-IDBS	C4-ODSS	C5-ODPS	C6-ODBS
0	0.00	2.44	0.31	1.91	− 9.89	− 6.23	− 9.57
4	0.00	4.29	− 1.79	1.68	− 5.36	− 5.09	− 6.81
8	0.00	6.23	− 1.72	2.84	− 3.80	− 4.51	− 5.13
12	0.00	**9.29**	− 3.56	2.84	− 2.55	− 4.91	− 3.50

Bold signifies the maximum drag reduction and L/D ratio enhancement achieved.

### Flow fields visualisation

**Figure 8:**
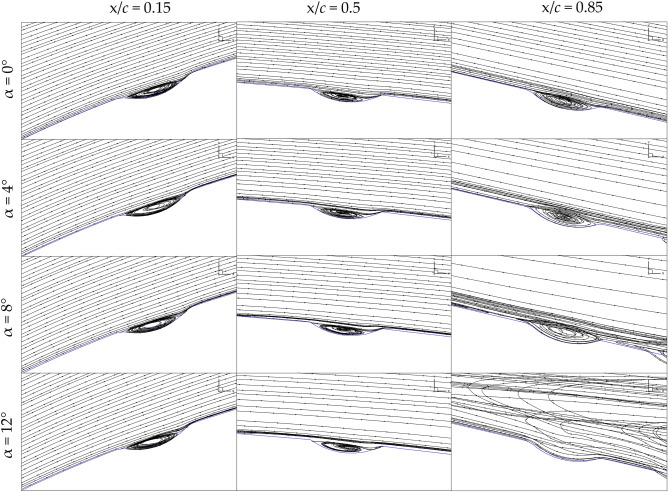
Velocity streamlines for C1-IDSS and flow behavior inside the dimples at all angles of attack.

Based on the findings from the preceding section, dimple configuration C1-IDSS emerges with the most substantial drag reduction, resulting in an increased L/D ratio compared to both the original wing and all other configurations. In order to explore further the influence of dimples on wing aerodynamics, the velocity streamlines at various chordwise Sections. (15%, 50%, and 85%) for this particular case are scrutinized across all angles of attack, as depicted in Fig. 8. The figure reveals that the flow field at 15% and 50% chordwise positions, for all angles of attack, exhibits similar flow behavior. The flow enters the dimples, circulates, and exits downstream of the dimples, maintaining attached flow to the wing surface without causing any flow separation. This observation indicates that the dimple cavity creates localized turbulence, which serves to energize the boundary layer, promoting adhesion between the flow and the wing surface. At 85% chord onwards, near the trailing edge, the turbulent boundary layer induced by the dimples delays the onset of flow separation. As the angle of attack increases, the dimples aid in maintaining attached airflow by preventing premature separation, a common issue in traditional wing designs. This delayed separation contributes to a reduction in drag, particularly advantageous in scenarios involving low-speed and high angles of attack. The analyses of the flow field align closely with the outcomes presented in Table 3. In essence, with proper design and optimization, the incorporation of dimples on wing surfaces proves to be a promising approach for enhancing aerodynamic performance.

### Surface-limited pathlines and pressure contours

**Figure 9:**
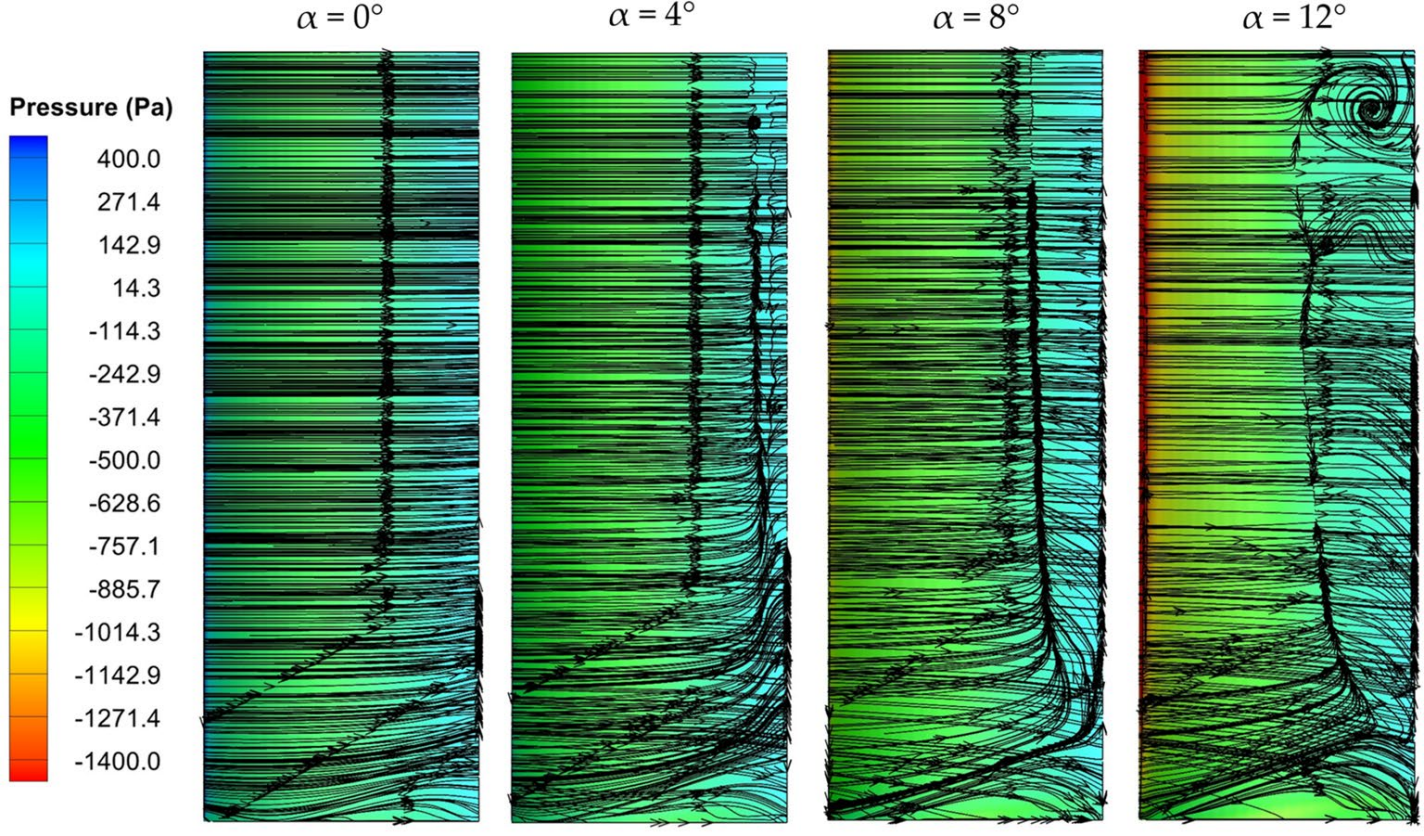
Surface-limited streamlines with pressure contour for baseline wing (no dimples).

**Figure 10:**
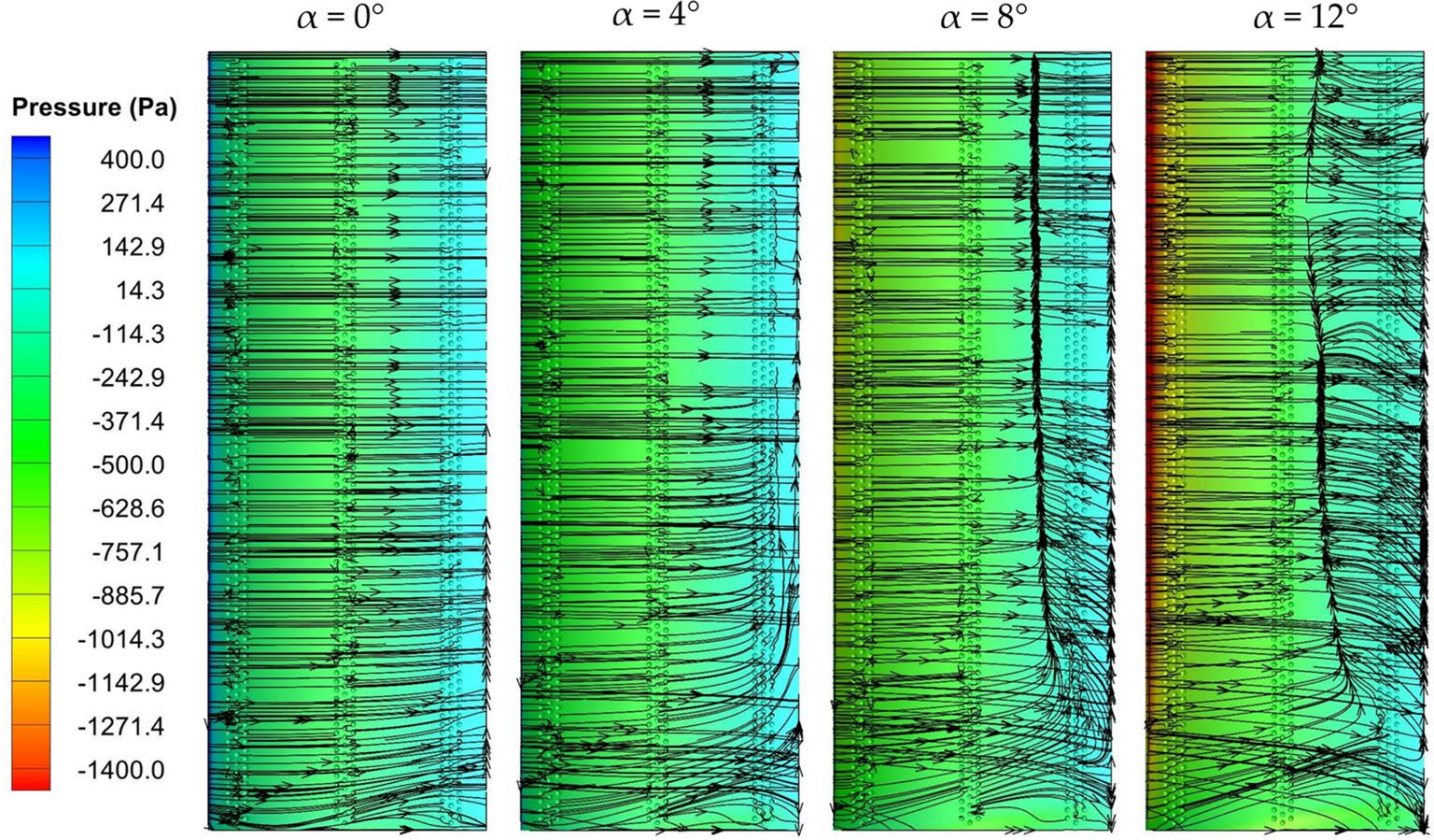
Surface-limited streamlines with pressure contour for C1-IDSS.

Analyzing the near-wall flow characteristics is instrumental in understanding the flow physics on the wing surface at varying angles of attack. Figures 9 and 10 demonstrate the surface-limited pathlines and pressure contours of the unmodified wing and dimple configuration C1-IDSS on the suction surface at all angles of attack. These pathlines assessment reveal the presence of vortex regions and reverse-flow zones that contribute to flow separation, providing insights into the flow phenomenon on the wing surface. The occurrence of flow separation on the wing becomes evident by the intersection of reverse streamlines, which reveals distinctive behaviours between the simple wing and the dimpled wing. For the no dimpled wing, the surface limiting pathlines exhibit an earlier deviation and separation from the wing surface as the angle of attack increases. This premature separation leads to increased drag, limiting the wing's overall performance. Conversely, the dimpled wing demonstrates a more favourable scenario. Dimples significantly alter the flow behaviour, creating localized turbulence at lower angles of attack that energizes the boundary layer, enhancing adhesion between the flow and the wing surface as also discussed in previous section. This effect is visually represented by surface limiting streamlines in Fig. 10 which maintain proximity to the wing, reducing the likelihood of premature separation.

The pressure contours further substantiate the effectiveness of dimples in delaying flow separation. In the case of the simple wing, pressure contours indicate a gradual decrease in pressure along the wing surface, leading to premature separation at higher angles of attack. This separation introduces turbulent airflow and increases drag. However, the dimpled wing shows pressure contours that illustrate a delayed onset of flow separation. The localized turbulence generated by the dimples contributes to a more gradual decrease in pressure, preserving the attached airflow at higher angles of attack.

In conclusion, streamline visualization and pressure contour analysis revealed that dimples on a wing demonstrably delay flow separation compared to a non-dimpled wing. This is attributed to turbulence generated by the dimples, which energizes the boundary layer and promotes better flow adhesion. As a result, the inward dimpled wing experiences a delayed stall and potentially reduced drag characteristics, suggesting its potential for enhanced overall wing performance.

In summary, the implementation of dimples on the wing surface augments the aerodynamic performance. Nevertheless, in line with other passive techniques for flow control, the utilization of dimples as a strategy for passively regulating flow fields on lifting surfaces lacks universal applicability. In general, this study, along with its counterparts, contributes to the ongoing exploration of sustainable and eco-friendly aviation. It serves as a pivotal stride in devising universally optimized aerodynamic configurations to benefit aviation industry across a diverse range of operational circumstances.

Considering that the incorporation of dimples leads to performance deterioration in certain configurations (outward dimples), it is imperative to conduct further exploration and optimization of dimple design parameters for enhanced flow control. Variables such as location of dimple, diameter, indentation depth, and dimple shape present avenues for exploration in subsequent research. As outlined in Section “Meshing and validation”, the discrepancy in drag coefficient between the medium and fine mesh remains below 0.7%. This value could be further minimized to achieve even higher accuracy in the results. However, the limited computational power in this study prevented achieving the lower margin of error, a challenge that could be addressed in future research.

In addition, it is acknowledged that the unstructured mesh and RANS model employed in this study have inherent limitations in predicting complex flow behaviors, particularly in regions of flow separation and recirculation, such as those within dimples. While RANS models offer valuable insights into the overall flow field, they may not fully capture small-scale turbulent structures and transient phenomena, as they rely on modeling assumptions to derive their mathematical formulations. Consequently, in future applications, it may be beneficial to consider employing structured meshing strategies and exploring the possibility of utilizing more comprehensive methods such as Large Eddy Simulation (LES) or Detached Eddy Simulation (DES) to better capture turbulent structures within dimples and assess their impact on aerodynamic performance.

From a manufacturing perspective, it is important to assess the viability of implementing dimples. This involves conducting a cost–benefit analysis and feasibility study to determine whether such an implementation is practical and worthwhile.

## Conclusion

This study investigates the impact of surface modification on a straight rectangular wing using dimples, strategically positioned on either the suction or pressure side, as well as on both sides. Specifically, six configurations of the dimpled-surface wing are studied with inward and outward spherical dimples at the free stream velocity of 32.15 m/s over the angle of attack ranging from 0 to 12°. The resolution of governing continuity and momentum equations is accomplished through an incompressible RANS solver, coupled with the *k—ω* (SST) turbulent model. To validate the simulation approach, comprehensive comparisons with experimental data and existing studies are conducted throughout the study. The results show that dimples have the potential to augment the aerodynamic characteristics of lifting surfaces, if they are designed appropriately. For instance, we observed a significant reduction of the drag coefficient, by approximately 6.6%, in the best-case scenario. Among the six dimple configurations, the configuration with inward dimples on the suction surface exhibited the best performance. Importantly, this improvement did not adversely impact the lift coefficient, resulting in an enhanced L/D ratio.

From a design perspective, the passive control of flow over lifting surfaces through dimples can substantially improve aerodynamic performance when correctly implemented. With the growing demand in air traffic and increasing environmental concerns, our proposed improvement aligns with the objective of achieving cleaner aviation with higher fuel efficiency.

While our research underscores the potential benefits of dimples in aerodynamics, it is important to acknowledge that certain assumptions and simplifications are involved in numerical simulations. An experimental wind tunnel test can take this concept to a practical level in shaping the future of aerodynamics and the aviation industry. Future research directions to refine the surface flow control may involve further optimizing dimple designs, including their shape, diameter, indentation depth, pitch distance and placement locations. Additionally, the implementation of a dimpled surface as active flow control, with the potential for automatic adaptation to diverse operating conditions, presents a promising avenue for future exploration.

## Data Availability

All data generated or analyzed during this study are included in this article. Also, they are available from the corresponding author upon request.
